# Causes of Death among AIDS Patients after Introduction of Free Combination Antiretroviral Therapy (cART) in Three Chinese Provinces, 2010–2011

**DOI:** 10.1371/journal.pone.0139998

**Published:** 2015-10-27

**Authors:** Liyan Wang, Lin Ge, Lu Wang, Jamie P. Morano, Wei Guo, Kaveh Khoshnood, Qianqian Qin, Zhengwei Ding, Dingyong Sun, Xiaoyan Liu, Hongbing Luo, Jonas Tillman, Yan Cui

**Affiliations:** 1 Division of Epidemiology, National Center for AIDS/STD Control and Prevention, Chinese Center for Disease Control and Prevention, Beijing, China; 2 University of South Florida, Morsani College of Medicine, Division of Infectious Disease and International Medicine, USF Medicine International, Tampa, Florida, United States of America; 3 Yale School of Public Health, New Haven, Connecticut, United States of America; 4 Henan Center for AIDS/STD Control and Prevention, Henan Center for Disease Control and Prevention, Henan, China; 5 Jiangsu Center for AIDS/STD Control and Prevention, Jiangsu Center for Disease Control and Prevention, Jiangsu, China; 6 Yunnan Center for AIDS/STD Control and Prevention, Yunnan Center for Disease Control and Prevention, Yunnan, China; 7 Division of Intervention, National Center for AIDS/STD Control and Prevention, Chinese Center for Disease Control and Prevention, Beijing, China; Istituto Superiore Sanita', ITALY

## Abstract

**Introduction:**

Although AIDS-related deaths have had significant economic and social impact following an increased disease burden internationally, few studies have evaluated the cause of AIDS-related deaths among patients with AIDS on combination anti-retroviral therapy (cART) in China. This study examines the causes of death among AIDS-patients in China and uses a methodology to increase data accuracy compared to the previous studies on AIDS-related mortality in China, that have taken the reported cause of death in the National HIV Registry at face-value.

**Methods:**

Death certificates/medical records were examined and a cross-sectional survey was conducted in three provinces to verify the causes of death among AIDS patients who died between January 1, 2010 and June 30, 2011. Chi-square analysis was conducted to examine the categorical variables by causes of death and by ART status. Univariate and multivariate logistic regression were used to evaluate factors associated with AIDS-related death versus non-AIDS related death.

**Results:**

This study used a sample of 1,109 subjects. The average age at death was 44.5 years. AIDS-related deaths were significantly higher than non-AIDS and injury-related deaths. In the sample, 41.9% (465/1109) were deceased within a year of HIV diagnosis and 52.7% (584/1109) of the deceased AIDS patients were not on cART. For AIDS-related deaths (n = 798), statistically significant factors included CD4 count <200 cells/mm^3^ at the time of cART initiation (AOR 1.94, 95%CI 1.24–3.05), ART naïve (AOR 1.69, 95%CI 1.09–2.61; p = 0.019) and age <39 years (AOR 2.96, 95%CI 1.77–4.96).

**Conclusion:**

For the AIDS patients that were deceased, only those who initiated cART while at a CD4 count ≥200 cells/mm3 were less likely to die from AIDS-related causes compared to those who didn’t initiate ART at all.

## Introduction

The AIDS epidemic is a serious global public health issue [1]. AIDS-related deaths have had a significant economic and social impact throughout the world, and have led to an increased disease burden, particularly in areas with limited health resources [2]. Combination antiretroviral therapy (cART) has been proven to be an effective way to reduce AIDS-related deaths and improve life expectancy for HIV patients [3–7]. Studies from highly heterogeneous study settings have shown that cART has decreased AIDS-related deaths to percentages close to or below rates of non-AIDS related deaths [8–9]. However, the results from some recent studies in China have showed that the proportion of AIDS-related deaths still is higher than non-AIDS related deaths, even among patient on cART [10–13].

China’s national cART program began in 1999, and in 2003 the "Four Frees and One Care" policy led to improved access to cART provided free-of charge [14]. In 2008, the second version of the “Free cART Guidelines” recommended to initiate cART at CD4 counts <200 cells/mm^3^, and this threshold for eligibility was further increased to <350 cells/mm^3^ in 2012 [14].

The positive impact of cART on reducing AIDS related deaths is well known. However, relatively few studies have examined AIDS-related deaths among HIV patients on cART in China. Furthermore, the existing studies have used data from the Chinese National HIV Registry. This data may suffer from misreporting, particularly in regard to cause of death. This study explored whether or not cART made a difference in the proportion of causes of death that were AIDS-related as compared to non-AIDS related, and also explored the factors related to dying from these causes using a sample including all Chinese HIV positive individuals who died in three geographical areas, spread over three Chinese provinces between January 1, 2010 to June 30, 2011. These areas were chosen to represent key drivers of the epidemic in China historically–injection drug use (IDU), blood plasma donation, and sexual transmission–and to investigate if there were any systematic differences among these highly differentiated demographics. Before 1995, the key driver of the epidemic was IDU. This was followed by former plasma donors (FPDs), commonly located in rural areas in central China, infected at mass as a result of clinical malpractices by private actors on the poorly regulated blood product market in the mid 1990’s [15]. This group, made up of mainly farmers in rural areas, show low levels of overlap with other modes of transmission used in this study and made up 10.7% of all people in China living with HIV in 2005 [16, 17]. Since 2005, the spread of the HIV epidemic is mainly driven by sexual transmission [18]. The data on cause of death was reclassified using death certificates, medical records and a household survey.

## Methods

### Participants

In order to evaluate if there are any systematic differences in the causes of death between deceased AIDS patients from areas with different key drivers of the epidemic in recent years, all reported deceased AIDS cases (CD4 <200 and/or showing clinical symptoms) in Dehong Prefecture in Yunnan Province, Zhumadian City in Henan Province, and the entire Jiangsu Province between January 1, 2010 and June 30, 2011 were included in the sample. These areas were chosen based on the understanding that the HIV epidemic in these areas have been primarily driven by a certain mode of transmission (IDU in Dehong, FPDs in Zhumadian and sexual transmission in Jiangsu Province). Even though FPD as a mode of transmission is negligible for newly infected cases in recent years, a significant proportion of people living with HIV in China were infected through plasma donation, and it is therefore of interest to investigate whether the causes of death differs for this subpopulation. The FPDs have been living with HIV a long time, many of them without access to treatment for years after being infected, and a significant proportion of these cases are in a stage of clinical AIDS.

### Materials

The National Center for AIDS/STD Control and Prevention (NCAIDS), Chinese Center for Disease Control and Prevention (China CDC) National Surveillance HIV/AIDS Reporting System (NSHRS) is a comprehensive internet-based database first used in 2005 that covers more than 3,000 counties. The lists of all deceased AIDS cases between January 1, 2010 and June 30, 2011 in the specified regions were downloaded from the database. The data included demographic information, history of HIV related risk behaviors, date of cART initiation, date of death, date of HIV diagnosis and CD4 count at cART initiation.

All diagnosed and confirmed HIV-infected cases in China are required by law to be registered in the National HIV Registry within 24 hours of detection, and all HIV positive individuals are required to go through regular follow-up medical examinations [19].

A detailed twenty nine-item questionnaire was developed specifically for this study in order to gather more complete information on the cause of death for the subjects. Additional questions included demographics, history of high-risk behaviors, and co-existing co-morbidities before time of death **(S1 Text)**.

**Fig 1:**
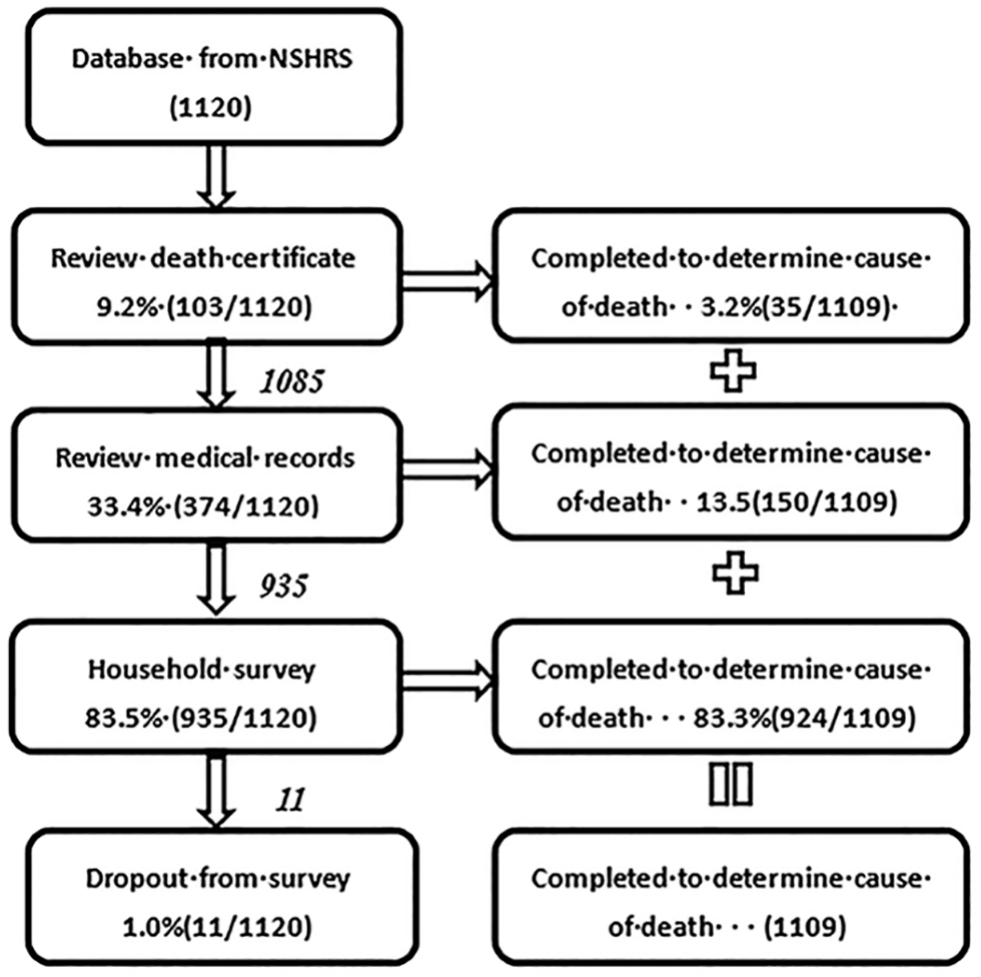
Flow chart of data collection procedure.

First, investigators completed a review of the death certificates for the subjects in the sample. Out of the 103 subjects (total N = 1,120) where the investigators could access the death certificates, only 35 were judged to be complete enough to accurately determine cause of death. For the remaining cases, investigators went on to review medical records to determine the cause of death. Out of 374 medical records that could be found for the remaining subjects without confirmed cause of death (n = 1,120–35 = 1,085), 150 were judged to be complete enough to accurately determine the cause of death. For the remaining 935 (1,120-35-150), personnel involved in the study proceeded to complete the household survey (Fig 1).

Health care workers conducted semi-structured face-to-face interviews, where they were instructed to follow the format of the survey for the interviews, with key informant family members, close friends, former partners or the village doctor in a private room after obtaining oral informed consent. The completed questionnaires were then used to determine cause of death. For the sample used for analysis (1,109), death certificates were used for 3.2% (35/1,109), medical records 13.5% (150/1,109) and informant data 83.3% (924/1,109) of the cases to determine cause of death.

All collection of medical information and household surveys were conducted by certified health professionals, who had provided healthcare services to the deceased AIDS patients and were familiar with the patient’s family members. These health professionals also spoke the local language in the regions where they conducted the survey.

### Coding of cause of death

In order to streamline the coding for cause of death in the subjects where questionnaires were used, ICD-10 coding with the Chinese Ministry of Health HIV Infection Diagnostic Criteria [20] and the U.S. CDC AIDS-Defining Conditions [21] were used to create standardized coding procedures. AIDS was defined as HIV positive individuals with a CD4 count <200 cells/mm^3^ confirmed with western blot testing or presence of opportunistic infections, in accordance to the Chinese Ministry of Health HIV Infection Diagnostic Criteria.

This coding included three main categories with 49 sub-categories: AIDS related death (30 sub-categories), non-AIDS related death (11 sub-categories), and injury related death (8 sub-categories). AIDS-related death was defined as opportunistic and parasitic infections, AIDS-related malignancy, and other AIDS specified disease syndromes, including encephalopathy, lymphoid interstitial pneumonia, generalized lymphadenopathy, or AIDS wasting syndrome as main cause of death. Non-AIDS related death was, in accordance with the ICD-10 coding, defined as digestive diseases (including hepatitis B or C), cardio-cerebrovascular disease and disease of respiratory, urogenital, endocrine, hematological, and central nerve systems. Deaths from drug overdose, poisoning, suicide, homicide, or fracture was categorized as injury related death. For the analysis in this paper only the main categories (AIDS-related, non-AIDS related and injury-related deaths) were used, but disaggregated figures can be found in S2 Table.

The final decision for cause of death for each AIDS subject was confirmed by members of investigation group who were trained in the standardized coding procedure and supervised by experts from NCAIDS. All causes of death were adjudicated by NCAIDS.

### Data Entry and Analysis

All survey data was entered and verified at the local CDC level in each of the three geographical areas and validated using Epidata 3.1 [22]. Further data analysis was conducted using SPSS18.0 for Windows [23].

The causes of death were investigated using three distinct methods. First,Chi-square was used to examine any differences in the proportion of cause of death (AIDS-related, non-AIDS related, and injury related) in the three regions. Second, Chi-square was used to examine any differences in the proportion of death by ART category: ART naïve (never received ART), ART initiated at CD4 count <200 cells/mm^3^, and ART initiated at CD4 count ≥200 cells/mm^3^. Third, stepwise univariate and multivariate logistic regression with significance criterion p<0.05 were used to construct the most robust correlates of cause of death among the subjects. A multivariate model where the outcome of AIDS-related death as compared to non-AIDS related death was constructed. Kaplan-Meier curves were constructed as a visual tool to show the time of death stratified by cART status.

### Quality Control

In order to complete the questionnaires, trained national NCAIDS CDC staff invited health care workers from both local hospitals and community clinics to provide preliminary information, and when needed, families and neighbors of the deceased were asked to provide supplementary information that could help determine cause of death.

Further assistance was obtained from specialists at the China CDC National Cause of Death Surveillance Department in Beijing and China CDC specialists on ART and AIDS epidemiology. To ensure follow-up and completion, staff from local CDCs in the three areas regularly checked the collected information on a daily to weekly basis to ensure progress and compliance. Data was first obtained on the local level and controlled for completeness and accuracy by NCAIDS CDC Staff in Beijing.

### Ethical Issues

Data downloading was in compliance with the Chinese national regulatory standards of surveillance data, and the research protocol was reviewed and approved under the supervision of the Institutional Review Board of National Centre for AIDS/STD Prevention and Control, China CDC (IRB00002276, FWA00002958). All personnel involved in the study were allowed to access data from the National HIV Registry using standard China CDC procedures. Oral informed consent was obtained from all informants during the household survey, and a field supervisor was present in the field and required to sign the questionnaire if oral informed consent was given. The procedure for obtaining oral consent was also approved by the Institutional Review Board of National Centre for AIDS/STD Prevention and Control, China CDC. All records with personal information were eliminated and de-identified before data analysis.

## Results

### Baseline Socio-demographic Characteristics

Of the 1,120 cases identified as deceased from the National HIV Registry, 1,109 cases had complete cause of death information from the procedure described in the methods section. In the sample 68.2% were male, 65.5% married, and the average age at time of death was 44.5 years (S1 Table). FPD was the most frequent reported mode of transmission (43.1%), followed by sexual transmission (35.6%), and IDU (16.2%). Approximately 41.9% (n = 465) died within the first year of HIV diagnosis and 63.0% of those deceased died at age 40 years or above. For the three cART categories (naïve, initiated cART therapy at CD4 counts <200 cells/mme^3^ and initiated cART therapy at CD4 counts ≥200 cells/mm^3^), all socio-demographic and behavior-related variables for the sample (gender, marital status, mode of transmission and region) were found to be statistically significant using Chi-squared tests (p<0.001) (S1 Table).

Of the full sample used, a majority of those deceased (52.7%) never initiated cART (S1 Table). Of the 47.3% who did initiate cART, 67.8% (356/525) had CD4 count <200 cells/mm^3^, and 32.2% (169/525) had CD4 cell count ≥200 cells/mm^3^ at time of ART initiation. Even with ART initiation, a significant proportion of deaths occurred within three month from ART initiation (9.7%, 51/525).

### Comparison of the Cause of Death to the National HIV Registry

The cause of death determined by the methodology in this study differed greatly from the data in the National HIV Registry. Only 69.1% of the study population had a registered cause of death that was consistent in the registry and the data obtained for this study through death certificate/medical record review and household survey ([690+49+27]/1,109, Table 1). The major discrepancy was deaths classified as AIDS-related in the registry, but classified as non-AIDS related in this study (14.6%, 162/1,109) and vice-versa (7.8%, 86/1,109).

**Table 1:** Comparison of cause of death, case reporting system and field survey.

		Field survey		
		AIDS-related	Non-AIDS related	Injury-related
Case reporting	AIDS-related	**690**	162	44
system	Non-AIDS related	86	**49**	22
	Injury (suicide/overdose)	9	1	**27**
	Not specified	13	4	2

### Causes of Death

A total of 1,109 subjects were successfully assigned a cause of death using the methodology of this study (**S2 Table**). AIDS related deaths accounted for 72.0% (n = 798), of which 57.6% (460/798) were classified as AIDS-related infections, 27.7% (221/798) as other AIDS specified diseases or syndromes, 7.3% (58/798) as AIDS related malignancy and 7.3% (59/798) could not be sub-classified. Non-AIDS related deaths accounted for 19.5% (216/1,109), of which 19.2% (63/216) were sub-classified as hepatitis B or C, 24.1% (52/216) other digestive diseases, and 24.1% (52/216) cardio-cerebrovascular disease. Injury related deaths accounted for 8.6% (95/1,109) of AIDS patients deceased during the sample period, of which the largest subcategory was physical injury (69.5%, 66/95) (**S2 Table**).

Among non-injury related deaths, AIDS-related deaths were significantly higher than non-AIDS related deaths across all three regions, with the highest measured proportion in Dehong (80.4%, n = 262), and the lowest in Jiangsu Province (74.5%, n = 123) (Table 2). There was no statistically significant difference between the proportions for cause of death across the three geographical regions (p = 0.323, Table 2).

**Table 2:** Causes of death reported in those diagnosed with AIDS by category across three Chinese regions, January 1, 2010—June 30, 2011, excluding injury-related deaths, N = 1,014.

Cause of death	Dehong (IDU)	Jiangsu (Sexual)	Zhumadian (FPD)	χ^2^	p-value
AIDS related	262(80.4)	123(74.5)	413(79.0)	2.262	0.323
Non-AIDS related	64(19.6)	42(25.5)	110(21.0)		
**Sub-total**	**326**	**165**	**523**		

Compared to subjects who were either cART naïve and those who initiated cART with CD4 count <200 cells/mm^3^, those initiating cART at CD4 counts ≥200 cells/mm^3^ exhibited a significantly lower proportion of AIDS-related deaths (χ^2^ = 11.13, p = 0.007), but no statistically significant difference in injury-related deaths (χ^2^ = 1.09, p = 0.58).

**Fig 2:**
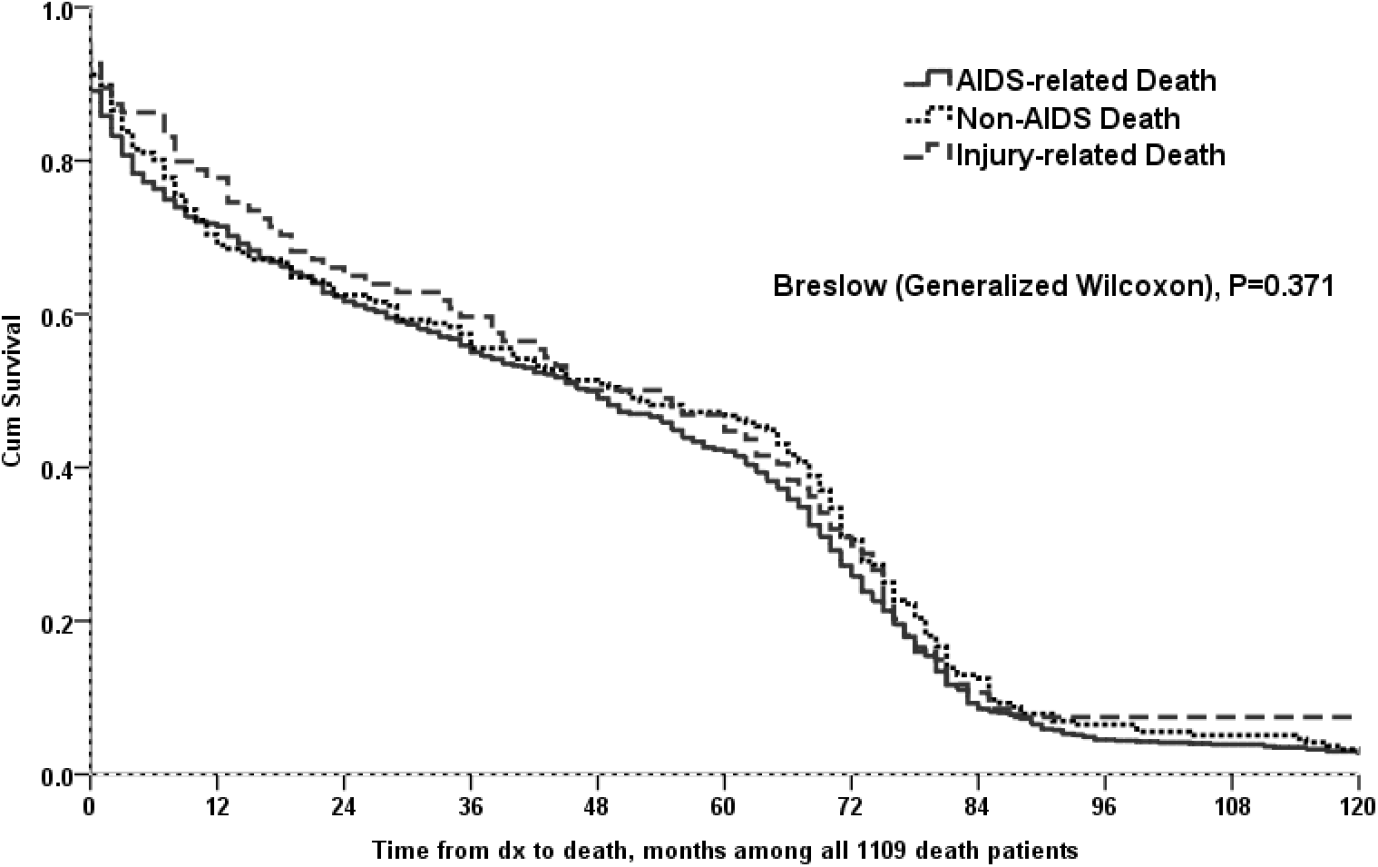
Time from diagnosis to death (months), among 1109 deceased patients (across different causes of death).

**Fig 3:**
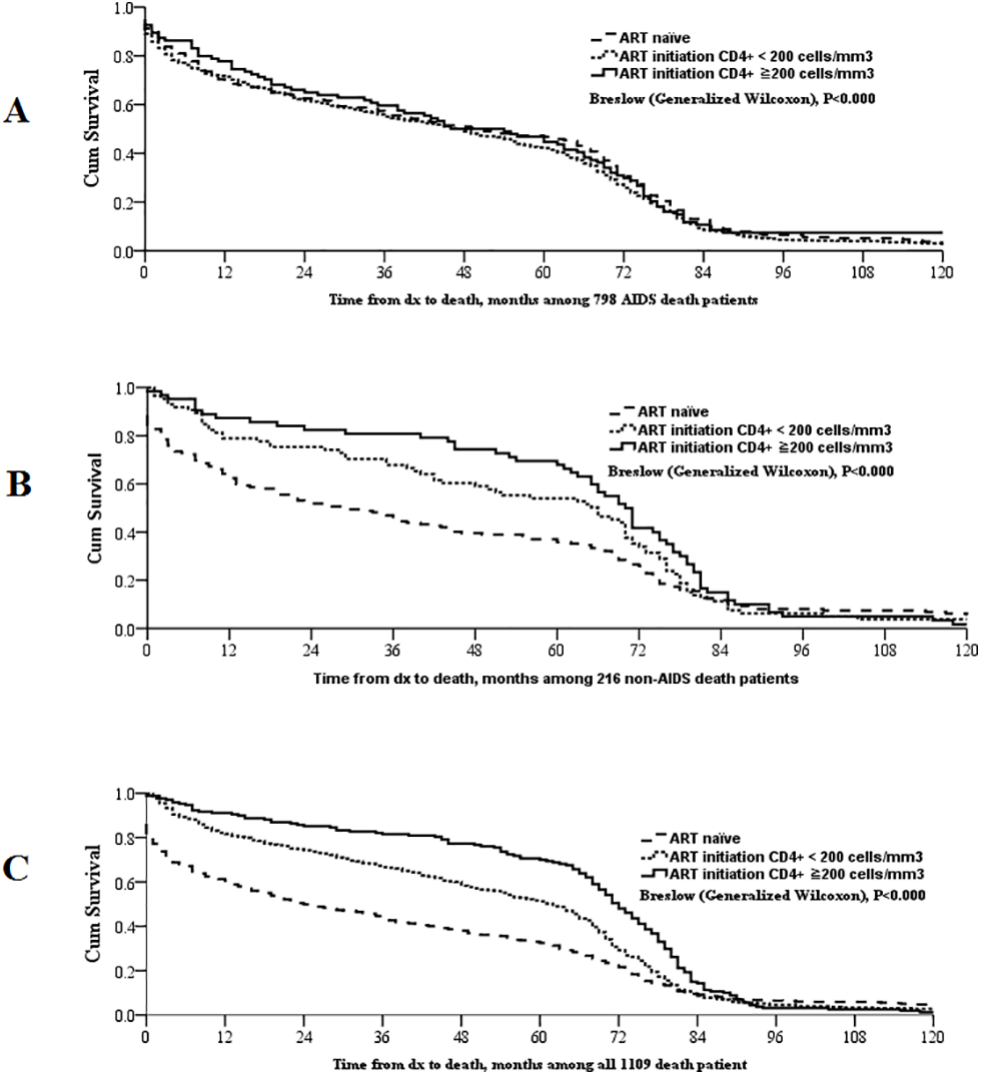
Time from diagnosis to death (months), across different cART groups, by cause of death (AIDS, non-AIDS, all causes). (A) Time from diagnosis to death (months), among 798 AIDS-related deaths; (B) Time from diagnosis to death (months), among 216 non-AIDS related deaths; (C) Time from diagnosis to death (months), among 1109 deceased patients; cART group was divided into ART naïve, ART initiation CD4 <200 cells/mm3 and ART initiation CD4 ≥200 cells/mm3.

Kaplan-Meier curves demonstrated that time to death was not a statistically significant variable for the sample (N = 1109) among cause of death (AIDS, non-AIDS, injury) (p = 0.371) (Fig 2). Among cART naïve subjects, the time from HIV diagnosis to death was shorter compared to both those subjects who initiated cART therapy, no matter the CD4 count (p<0.0001), as well as the subpopulation that initiated cART with CD4 count <200 cells/mm^3^ (p<0.0001). This was also true for non-AIDS related deaths (Fig 3).

The average time to death from HIV diagnosis was 37.3 months for ART-naïve subjects, 52.8 months for those who initiated ART with CD4 count <200 and 65.5 months for those who initiated ART with CD4 count ≥200.

### Correlates of Cause of Death

Given that we did not find a significant difference between the proportions for cause of death across the three geographical regions (p = 0.32, Table 2), the data for the regions were collapsed for the regression analysis.

Multivariate modeling included demographic factors such as gender, marital status, age at death, age at diagnosis, time from diagnosis to death, HIV transmission mode, and CD4 cell count level at time of cART initiation. In the final model, statistically significant factors for AIDS-related death, as compared to non-AIDS related deaths, included initiation of ART at CD4 count <200 cells/mm^3^ with AOR 1.94 (95%CI 1.24–3.05; p = 0.004), ART naïve AOR 1.69 (95%CI 1.09–2.61; p = 0.019) and age <39 with AOR 2.96 (95%CI 1.77–4.96; p = 0.001) (Table 3).

**Table 3:** Final Multivariate Model of HIV/AIDS-related deaths (N = 1109).

	AOR(95%CI)	p-value
AIDS-related death vs non-AIDS death		
ART category		
CD4 ct <200 cells/ml at ART initiation	1.94 (1.24–3.05)	0.004
ART naïve	1.69 (1.09–2.61)	0.019
CD4 ct ≥200 cells/ml at ART initiation	reference	
Age at death (Years)		
≤39	2.96 (1.77–4.96)	0.001
40–59	1.55 (0.98–2.45)	0.058
≥60	reference	

## Discussion

In this study, we demonstrated that in for the subsample of deceased AIDS patients in three Chinese provinces, only those who initiated cART while at a CD4 count ≥200 cells/mm3 were less likely to die from AIDS-related causes compared to those who didn’t initiate ART at all. However, this could partly be due to the fact that the group of cART naïve subjects might have had a wide range of CD4 counts. Previous studies have shown that cART reduces HIV-associated mortality. Chinana et al. showed that the proportion of total deaths caused by AIDS in Malawi fell from 42% to 17% due to cART [24], and Brinkhof et al. demonstrated that the introduction of cART decreased excess mortality among HIV-infected individuals to levels similar to the general population in sub-Saharan Africa [25].

Despite attempts to provide free cART to those eligible in China, this study shows that for this sample, 52.7% of deceased AIDS patients were not on cART, and 57.4% of those passed away within one year of being diagnosed with HIV. The corresponding figure for the full sample was 41.9%. Kaplan Meier curves showed that time to death occurred later for the subpopulation where cART was initiated at CD4 count ≥200 cells/mm^3^, compared to the subjects that initiated cART at CD4 count <200 cells/mm3 and those that were cART naïve. These results highlight the importance for further efforts to increase the proportion of early-diagnosed cases. This study was conducted before the cut-off level for cART eligibility was increased to CD4 count ≥350 cells/mm^3^ in 2008 but ought to still be relevant given that the antiviral drugs and protocol used in China have not been changed after this policy change, and that late diagnosis still is a salient issue in many parts of the country [26].

The proportion of injury-related deaths in the sample is approximately at the same level as for the general Chinese population [27]. For all subjects with AIDS, the proportion of injury-related deaths were not influenced by cART initiation.

Despite differences in the main mode of HIV transmission for the three geographical regions, we found uniformly high AIDS-related deaths and no significant difference in outcomes between the three regions or modes of transmission.

AIDS-related deaths, as compared to non-AIDS related deaths, were more common for individuals aged 39 or below. This is may be due to the choice of sample and the behavioral differences these groups are likely to exhibit.

Many of the older subjects have been diagnosed for a long time (33.9% aged >39 and 26.1% aged ≤39 were diagnosed at least 5 years ago), making this a subsample of individuals who have been living with HIV for an extended period of time. It is therefore likely that they lead distinctively different lives from the subjects who have been diagnosed more recently, and this older subsample are also likely exhibit higher rates of cART adherence. A large proportion of the older individuals in the sample were also from the FPD group. This group has been thoroughly screened, and thus, those living with HIV/AIDS are more likely to be on cART (FPD 68.4%, IDU 25.6%, sexual transmission 35.2%). Additionally, since older age is more commonly associated with all causes of death, many non-AIDS related causes of death are more common for this age group.

Lastly, this study has given indications that a large proportion of causes of death may be misreported in the National HIV Registry. If this is the case not only in these isolated geographical areas, it could lead to biased results in studies on AIDS-related mortality in China, particularly if the misreporting were systematic in any way (e.g. if some regions over-report AIDS-related deaths, while another region consistently under-reports this class). Against this background, it may be of relevance to provide further training for local CDC staff to improve the mortality data quality.

## Limitations

First, as some causes of death were not available in medical records, we commonly had to rely on information reported by family members or neighbors. Although recall bias or incomplete information could be an issue, trained CDC staff made efforts to follow-up with physicians or health care workers to confirm records, and we find no reason why this should bias the study in any particular way either by geographic region or by cause of death. We also confirmed accounts in the field and reconfirmed with available sources to obtain maximum accuracy. Second, even though medical records were thoroughly reviewed, some records might have been incomplete or inaccurate and some factors that could have influenced the cause of death might not have been recorded and thus omitted in this study. Third, patients included in the study that were reported to be on cART might have exhibited varying levels of medication compliance and been on different cART regimens that might have influenced mortality outcomes [28–30]. However, given the close follow-up by CDC on HIV positive patients, we believe that this is less of a concern for the current study. Lastly, since the sample only included those AIDS patients registered in the National HIV Registry, there is a risk of under-reporting for those who died without their sero-status being known.

## Supporting Information

S1 Table Baseline characteristics of deaths reported in persons with AIDS in three selected regions, January 1, 2010—June 30, 2011, China, N = 1109. 14 patients had age <15 years.
(DOCX)

S2 Table Causes of death reported in those living with AIDS by ART initiation category, January 1, 2010—June 30, 2011, China, N = 1109. Digestive Disease Statistics do not include subcategories of hepatitis B & C.
(DOCX)

S1 Text Questionnaire for this study. (DOCX)
