# Supplementary material for: Causes of Death among AIDS Patients after Introduction of Free Combination Antiretroviral Therapy (cART) in Three Chinese Provinces, 2010–2011
Source: PLoS One. 2015 Oct 27;10(10):e0139998. doi: 10.1371/journal.pone.0139998 (PMC4624241; doi:10.1371/journal.pone.0139998)
Supplement: S1 Table — 14 patients had age <15 years. (DOCX) [file pone.0139998.s001.docx]

Characteristics of subjects included in this analysis are summarized in Supplemental Table 1.

S1 Table．Baseline characteristics of deaths reported in persons with AIDS in three selected regions, January 1, 2010 - June 30, 2011, China, N=1109.

| Characteristics | Total | ART naïve |  | ART initiation CD4+ < 200 cells/mm^3^ |  | ART initiation CD4+ ≥200 cells/mm^3^ | χ^2^ | p-value |
| --- | --- | --- | --- | --- | --- | --- | --- | --- |
|  | N=1109 | N=584 |  | N=356 |  | N=169 |  |  |
|  | n(%) | n (%) |  | n (%) |  | n (%) |  |  |
| **Gender** |  |  |  |  |  |  | 16.427 | p<0.001 |
| Male | 756(68.2) | 427(73.1) |  | 231(64.9) |  | 98(58.0) |  |  |
| Female | 353(31.8) | 157(26.9) |  | 125(35.1) |  | 71(42.0) |  |  |
| **Age at diagnosis** |  |  |  |  |  |  | 18.438 | p<0.001 |
| ≤39 | 564(50.9) | 324(55.5) |  | 174(48.9) |  | 66(39.1) |  |  |
| 40-59 | 466(42.0) | 215(36.8) |  | 162(45.5) |  | 89(52.7) |  |  |
| ≥60 | 79(7.1) | 45(7.7) |  | 20(5.6) |  | 14(8.3) |  |  |
| **Mean age at death, (years)（SD）** | 44.5 (12.6) | 42.4（13.3） |  | 45.8（11.3） |  | 49.1（11.1） |  | p<0.001 |
| **Age（years）at death** |  |  |  |  |  |  | 37.114 | p<0.001 |
| ≤39 | 410(37.0) | 262(44.9) |  | 110(30.9) |  | 38(22.5) |  |  |
| 40-59 | 581(52.4) | 264(45.2) |  | 208(58.4) |  | 109(64.5) |  |  |
| ≥60 | 118(10.6) | 58(9.9) |  | 38(10.7) |  | 22(13) |  |  |
| **Marital status** |  |  |  |  |  |  | 25.927 | p<0.001 |
| Married | 726(65.5) | 353(60.4) |  | 257(72.2) |  | 116(68.6) |  |  |
| Divorced/widowed | 232(20.9) | 125(21.4) |  | 66(18.5) |  | 41(24.3) |  |  |
| Single | 151(13.6) | 106(18.2) |  | 33(9.3) |  | 12(7.1) |  |  |
| **Mode of transmission** |  |  |  |  |  |  | 166.32 | p<0.001 |
| IDU | 180(16.2) | 134(22.9) |  | 31(8.7) |  | 15(8.9) |  |  |
| Sexual | 395(35.6) | 256(43.8) |  | 108(30.3) |  | 31(18.3) |  |  |
| Formal blood donation | 478(43.1) | 151(25.9) |  | 206(57.9) |  | 121(71.6) |  |  |
| Other | 56(5.0) | 43(7.4) |  | 11(3.1) |  | 2(1.2) |  |  |
| **Time from diagnosis to death (years)** |  |  |  |  |  |  | 153.01 | p<0.001 |
| <1 | 465(41.9) | 335(57.4) |  | 96(27.0) |  | 34(20.1) |  |  |
| 1-4 | 249(22.5) | 115(19.7) |  | 106(29.8) |  | 28(16.6) |  |  |
| ≥5 | 395(35.6) | 134(22.9) |  | 154(43.3) |  | 107(63.3) |  |  |
| **Time from ART initiation to death (months)** |  |  |  |  |  |  | 34.184 | p<0.001 |
| <3 | 51(9.7) | - |  | 45(12.6) |  | 6(3.6) |  |  |
| 3-12 | 86(16.4) | - |  | 67(18.8) |  | 19(11.2) |  |  |
| 13-48 | 156(29.7) | - |  | 113(31.7) |  | 43(25.4) |  |  |
| 49-72 | 122(23.2) | - |  | 77(21.6) |  | 45(26.6) |  |  |
| ≥73 | 110(21.0) | - |  | 54(15.2) |  | 56(33.1) |  |  |
| **Regions** |  |  |  |  |  |  | 142.4 | p<0.001 |
| Yunan (IDU) | 369(33.3) | 256(43.8) |  | 83(23.3) |  | 30(17.8) |  |  |
| Henan (FPD) | 560(50.5) | 201(34.4) |  | 225(63.2) |  | 134(79.3) |  |  |
| Jiangsu (Sexual) | 180(16.2) | 127(21.7) |  | 48(13.5) |  | 5(3.0) |  |  |

*Note: 14 patients had age < 15 years.
