# Supplementary material for: Causes of Death among AIDS Patients after Introduction of Free Combination Antiretroviral Therapy (cART) in Three Chinese Provinces, 2010–2011
Source: PLoS One. 2015 Oct 27;10(10):e0139998. doi: 10.1371/journal.pone.0139998 (PMC4624241; doi:10.1371/journal.pone.0139998)
Supplement: S2 Table — Digestive Disease Statistics do not include subcategories of hepatitis B & C. (DOCX) [file pone.0139998.s002.docx]

Causes of death by ART initiation category are shown in Supplemental Table 2.

**S2 Table． Causes of death reported in those living with AIDS by ART initiation category, January 1, 2010 - June 30, 2011, China, N=1109.**

| CDC NCAIDS Death Code Category (based on ICD-10) | Total |  | ART naïve |  | ART initiation  CD4+ <200 cells/mm^3^ |  | ART initiation  CD4+ ≥200  cells/mm^3^ | p-value |
| --- | --- | --- | --- | --- | --- | --- | --- | --- |
|  | N=1109  n (%) |  | N=584  n (%) |  | N=356  n (%) |  | N=169  n (%) |  |
| Non-injury Deaths | **1014(91.4)** |  | **530(90.8)** |  | **330(92.7)** |  | **154(91.1)** | 0.580 |
| **1 AIDS** | 798(72.0) |  | 422(72.3) |  | 270(75.8) |  | 106(62.7) | 0.007^*^ |
| 1.1 AIDS related infection (including opportunistic infections) | 460(41.5) |  | 218(37.3) |  | 175(49.2) |  | 67(39.6) | <.001^*^ |
| 1.2 AIDS related malignancy | 58(5.2) |  | 32(5.5) |  | 18(5.1) |  | 8(4.7) | 0.914 |
| 1.3 AIDS specified disease/ syndrome, | 221(19.9) |  | 122(20.9) |  | 69(19.4) |  | 30(17.8) | 0.635 |
| not further specified | 59(5.3) |  | 50(8.6) |  | 8(2.2) |  | 1(0.6) | <.001^*^ |
| **2 Non-AIDS** | 216(19.5) |  | 108(18.5) |  | 60(16.9) |  | 48(28.4) | 0.005^*^ |
| 2.1 Digestive diseases* | 52(4.7) |  | 32(5.5) |  | 12(3.4) |  | 8(4.7) | 0.333 |
| 2.1.1 Hepatitis B or C | 63(5.7) |  | 28(4.8) |  | 16(4.5) |  | 19(11.2) | 0.003^*^ |
| 2.2 cardio-cerebrovascular disease | 52(4.7) |  | 24(4.1) |  | 15(4.2) |  | 13(7.7) | 0.133 |
| 2.3 respiratory disease | 18(1.6) |  | 11(1.9) |  | 6(1.7) |  | 1(0.6) | 0.614 |
| 2.4 urogenital disease | 8(0.7) |  | 3(0.5) |  | 3(0.8) |  | 2(1.2) | 0.564 |
| 2.5 endocrine system diseases | 10(0.9) |  | 3(0.5) |  | 5(1.4) |  | 2(1.2) | 0.276 |
| 2.6 hematological diseases | 4(0.4) |  | 2(0.3) |  | 1(0.3) |  | 1(0.6) | 0.641 |
| 2.7 central nervous system disease | 4(0.4) |  | 3(0.5) |  | 0(0) |  | 1(0.6) | 0.378 |
| 2.8 other diseases | 4(0.4) |  | 2(0.3) |  | 1(0.3) |  | 1(0.6) | 0.641 |
| not further specified | 1(0.1) |  | 0(0) |  | 1(0.3) |  | 0(0) | 0.473 |
| **Injury deaths** | **95(8.6)** |  | **54(9.2)** |  | **26(7.3)** |  | **15(8.9)** | 0.580 |
| 3 suicide | 25(2.3) |  | 12(2.1) |  | 9(2.5) |  | 4(2.4) | 0.852 |
| 4 physical injury and poison/overdose | 66(6.0) |  | 41(7.0) |  | 16(4.5) |  | 9(5.3) | 0.264 |
| 5 other violent death | 4(0.4) |  | 1(0.2) |  | 1(0.3) |  | 2(1.2) | 0.142 |

* Digestive Disease Statistics do not include subcategories of hepatitis B & C.
