# Supplementary material for: Causes of Death among AIDS Patients after Introduction of Free Combination Antiretroviral Therapy (cART) in Three Chinese Provinces, 2010–2011
Source: PLoS One. 2015 Oct 27;10(10):e0139998. doi: 10.1371/journal.pone.0139998 (PMC4624241; doi:10.1371/journal.pone.0139998)
Supplement: S1 Text — (DOCX) [file pone.0139998.s003.docx]

**Questionnaire**

1. **Basic information**

A1. Name：

A2.Gender：① Male ② Female

A3.Nationality: ① Han ② Other: _________

A4.Occupation：

A5.Marital status：① Single ② Married ③ Divorced/widowed ⑨ Unknown

A6.Date of death： year month day

A7.Site of death：

① Hospital ward/emergency room ② At home/on the way to hospital ③ Not within the county of residence ④ Town/village hospitals ⑤ Nursing homes ⑥ Community hospitals or clinics ⑦Other: _________ ⑧ Unknown

**B. HIV/AIDS related diseases and symptoms**

B01. Which of the following deceased AIDS-related opportunistic infections were the deceased suffering from (multiple choice)：

① Cytomegalovirus infection (herpes simplex virus) ② Pneumocystis carinii pneumonia (PCP) ③ Cryptococcal meningitis ④ Infections located in the mouth and/or esophagus candidiasis ⑤ Toxoplasmosis ⑥ Mycobacterial infections (tuberculosis) ⑦ Others: _________ ⑧ None of the above

B02. Which of the following AIDS-related malignancy were the deceased suffering from (multiple choice)：

① Kaposi's sarcoma ② Burkitt's lymphoma ③ Primary effusion lymphoma ④Hodgkin's lymphoma ⑤ Others: _________ ⑥ None of the above

B03. Which of the following AIDS-related disease syndromes were the deceased suffering from (multiple choice)：

① AIDS-related encephalopathy (dementia ADC) ② Lymphoid tissue interstitial pneumonia ③ Wasting syndrome ④ Acute HIV infection syndrome ⑤ Generalized lymphadenopathy ⑥ AIDS-related blood diseases ⑦ Inflammatory immune reconstitution syndrome (IRIS) ⑧ Others: __________ ⑨ None of the above

B04. Did the deceased suffer from any additional diseases? (multiple choice)：

① HBV ② HCV ③ Hemophilia A ④ Mycobacterial infection ⑤Others: ______ ⑥None of the above

B05. Did the deceased receive antiviral therapy：① Yes ② No (Skip to C01)

B06. Did the deceased suffer from any of the following cART-related symptoms or diseases? (multiple choice)：

① cART-related diabetes ② cART-related pancreatitis ③ cART-related lipid metabolism ④ cART-related Hypertension ⑤ Others: _________ ⑥ None of the above

**C. List all other non-AIDS related diseases and symptoms the deceased were suffering from:**

C01.

C02.

C03.

C04.

C05.

**D.** Cause of death inference

D01. Sources of information collection (multiple choice)：

① Hospital records ② Outpatient Record ③ Autopsy Report ④ Medical death certificate ⑤ Clinicians ⑥ Township/town/village hospitals doctors ⑦ Family/friends of patients ⑧ Other: ___________

D02. Highest administrative diagnostic unit for diseases other than HIV/AIDS：

① Provincial (municipal) hospital ② Regional level (city) hospital ③ County (district) hospital ④ Township hospital ⑤ Village hospital ⑥ No official diagnosis ⑦ Forensic ⑧ Other: ___________

D03. The deceased were suffering from occupational diseases：① Yes ② No (skip to D04)

D03a. If yes, these were (list):_____________

D04. Was the death of the deceased sudden? ① Yes ② No (skip to D05)

D04a. If yes，the cause of death was: _____________

D05. Was the death of the deceased accidental? ① Yes ② No (skip to D06)

D05a. If yes，the cause of death was: _____________

D06. Did the deceased commit suicide? ① Yes ② No

D07. Was the cause of death due to poisoning? ① Yes ② No (skip to D08)

D07a. If yes, list the poisonous items/chemicals _____________

D08. Before the death of the deceased, which clinical manifestations and symptoms were present (multiple choice):

① Respiratory, circulatory failure ② Gastrointestinal bleeding ③ Multiple organ dysfunction (MODS) ④ Cancer cachexia ⑤ Acid-base imbalance ⑥ Electrolyte imbalance ⑦ Systemic failure ⑧ Massive hemoptysis ⑨ Pulmonary encephalopathy ⑩ Others: ___________

D09.The main diagnosis of death (Please fills in the name of a specific disease, do not fill in symptoms)

D09a Direct/immediate cause of death (disease)：__________________

D09b Underlying cause of death (disease)：__________________
D10. Death classified as (fill in underlying cause of death disease coding, D09a)

D11. Was the cause of death AIDS-related?

① Yes ② No ③ Not enough information

(End of questionnaire)
